# *Anaplasma phagocytophilum* from Rodents and Sheep, China

**DOI:** 10.3201/eid1605.091293

**Published:** 2010-05

**Authors:** Lin Zhan, Wu-Chun Cao, Jia-Fu Jiang, Xiao-Ai Zhang, Yun-Xi Liu, Xiao-Ming Wu, Wen-Yi Zhang, Pan-He Zhang, Chang-Ling Bian, J. Stephen Dumler, Hong Yang, Shu-Qing Zuo, Chen-Yi Chu, Wei Liu, Jan H. Richardus, J. Dik F. Habbema

**Affiliations:** Beijing Institute of Microbiology and Epidemiology, Beijing, People’s Republic of China (L. Zhan, W.-C. Cao, J.-F. Jiang, X.-A. Zhang, X.-M. Wu, W.-Y. Zhang, P.-H. Zhang, C.-L. Bian, H. Yang, S.-Q. Zuo, C.-Y. Chu, W. Liu); Chinese People’s Liberation Army General Hospital, Beijing (Y.-X. Liu); The Johns Hopkins University School of Medicine, Baltimore, Maryland, USA (J.S. Dumler); Erasmus University Medical Center, Rotterdam, the Netherlands (J.H. Richardus, J.D.F. Habbema)

**Keywords:** *Anaplasma phagocytophilum*, isolation, rodents, sheep, China, rickettsia, research

## Abstract

To characterize the strains of *Anaplasma phagocytophilum* in wild and domestic animals in China, we isolated the organism from rodents and sheep in northeastern China. We isolated 3 strains (2 from rodents and 1 from sick sheep) through propagation in BALB/c mice and then cell culture in HL60 cells. The 3 isolates were identified by Wright-Giemsa staining, immunofluorescence, and electronic microscopy and were characterized by sequence analyses of the 16S rRNA gene, partial citrate synthase gene, major surface protein 4 gene, and heat shock protein gene. The multiple sequences of the 3 isolates were identical to each other but different from all known strains from other countries. The public health and veterinary relevance of the isolates deserves further investigation.

## Abstract 2

Three strains were isolated and characterized.

*Anaplasma phagocytophilum* has been recognized as an animal pathogen and is an emerging human pathogen of public health relevance. From 1994 to 2005, ≈3,000 cases of human granulocytic anaplasmosis were diagnosed in the United States (*1*), and in more recent years, sporadic and clustered cases have been reported in Europe and the People’s Republic of China (*2**–**5*). Human are usually infected by tick bites, although perinatal transmission or transmission through contact with infected animal blood has been reported (*1*). A broad variety of animal species are known to carry *A. phagocytophilum,* and humans are incidental dead-end hosts (*6*).

Various *A. phagocytophilum* strains have been isolated from humans (*6*), domestic and wild animals, and ticks in the United States and Europe (*1**,*^6^*,**7*). Prior serologic and molecular evidence suggests that *A. phagocytophilum* has also infected humans, rodents, and ticks in many Asian countries, including China, Japan, and Korea (*8**–**12*). Our objectives were to obtain isolates of *A. phagocytophilum* in vitro by using the HL60 cell line and to characterize the strains from wild and domestic animals in China.

## Materials and Methods

### Collection and Preparation of Specimens

In May 2009, live rodents were captured in wire mesh traps in the hinterland of the Changbai Mountains (42°45′N, 130°35′E) in Jilin Province, China, where natural infections with *A. phagocytophilum* in ticks and rodents have been reported (*8*). After their species and sex were identified, trapped rodents were euthanized and anatomized. The spleen was removed from each rodent and ground with sterile normal saline. Four dying sheep were found at the same site at the same time; blood samples were aseptically collected into tubes containing EDTA-K^2+^.

### Propagation of *A. phagocytophilum* in BALB/c Mice

For isolation of *A. phagocytophilum*, the spleen suspensions of the rodents were pooled into 12 groups according to species, and 0.3 mL of spleen suspension was intraperitoneally injected into 48 BALB/c mice (4 in each group). Blood samples from the 4 sheep were also pooled and injected into a group of BALB/c mice by the same means. After 7–14 days, blood samples were collected from each inoculated mouse and evaluated for infection by real-time PCR. All animal experiments were performed according to the approved Institutional Animal Care and Use Committee guidelines.

### Isolation of *A. phagocytophilum* in HL60 Cells

The HL60 leukemia cell line was used to cultivate *A. phagocytophilum* as described (*13*). A volume of 100–300 μL blood (in EDTA-K^2+^) from infected BALB/c mice was inoculated into HL60 cells at densities of 2 × 10^5^ to 6 × 10^5^ cells/mL (*13*).

### Wright-Giemsa Staining and Immunofluorescence Microscopy

Slides of peripheral blood or the cultured cells were stained with Wright-Giemsa (BaSO DIAGNOSTICS, INC, Zhuhai, China). An indirect immunofluorescence assay was performed after the slides of culture cells were fixed for 10 minutes in a 1:1 solution of methanol and acetone as described (*13*). Horse anti–*A. phagocytophilum* serum (kindly provided by Jenet E. Foley, University of California, Davis, CA, USA) and fluorescein isothiocyanate–conjugated goat antihorse immunoglobulin G (Zhongshan Biotechnique, Inc., Beijing, China) were used for the assay. Serum samples from healthy horses were used as negative controls.

### Electronic Microscopy

Infected HL60 cells were processed as previously described (*14*). Electron microscopic examination was conducted by using a Tecnai 10 electron microscope (Philips, Amsterdam, the Netherlands).

### PCR and Sequence Analysis

Real-time PCR selective for the major surface protein 2 gene (*msp2*) was used as described by Drazenovich et al. (*15*). To characterize the *A. phagocytophilum* strains isolated in the study, we amplified, purified, sequenced, and compared the 16S rRNA gene (*rrs*), partial sequences of the citrate synthase gene (*gltA)*, major surface protein 4 gene (*msp4*), and heat shock protein gene (*groEL*) as described (*8**,**16**,**17*). Phylogenetic analyses were performed and phylogenetic trees were constructed by using Mega 3.0 software (*17**,**18*).

### Nucleotide Sequence Accession Numbers

The nucleotide sequences of *A. phagocytophilum* isolated in this study were deposited in GenBank. Accession numbers are GQ412337–GQ412339 for 1,431-bp *rrs*, GQ412340–GQ412342 for 348-bp to 348-bp *gltA*, GQ412343–GQ412345 for 428-bp *groEL*, and GQ412346–GQ412348 for 779-bp *msp4*.

## Results

### *A. phagocytophilum* in BALB/c mice

**Figure 1:**
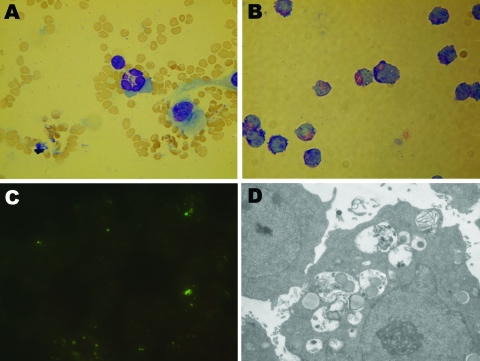
Photomicrographs of cells infected with *Anaplasma phagocytophilum*. A) Wright-Giemsa–stained granulocytic cell of a BALB/c mouse. B) Wright-Giemsa-stained HL60 cells. C) Immunofluorescent-stained infected HL60 cells. D) Electron photomicrographs of an HL60 cell. Original magnifications ×1,500 (A–B), ×1,000 (C), and ×6,200 (D).

A total of 47 live rodents—20 black-striped field mice (*Apodemus agrarius*) and 27 great long-tailed hamsters (*Tscherskia triton*)—were captured. When tested 7–14 days postinoculation, every mouse in 5 of the 12 groups of inoculated BALB/c mice was positive for *A. phagocytophilum* according to real-time PCR selective for the *msp2* gene; 3 groups were *A. agrarius* mice and 2 were *T. triton* hamsters. Two BALB/c mice inoculated with the anticoagulated blood samples from the 4 sick sheep were positive for *A. phagocytophilum* according to PCR. Typical morulae were observed in granulocytes of experimentally infected BALB/c mice (Figure 1, panel A).

### *A. phagocytophilum* in HL60 cells

Three *A. phagocytophilum* strains were propagated in HL60 cells: 1 from *A. agrarius* mice was named China-C-Aa, 1 from *T. triton* hamsters was named China-C-Tt, and 1 from sheep was named China-C-Y. *A.* *phagocytophilum* was first observed in Wright-Giemsa stain preparations 5 days after preparation of cultures (Figure 1, panel B). Morulae were found in ≈70% of HL60 cells at 10 days postinoculation. PCR showed all 3 agents cultured to be *A. phagocytophilum.* Blank control cultures (HL60 cells only) and cultures inoculated with blood of uninfected BALB/c mice showed no evidence of infection by Wright-Giemsa stain or PCR. Immunofluorescence microscopy demonstrated specific staining of *A. phagocytophilum* in infected cells (Figure 1, panel C). Such staining was not observed in uninfected cells or in cells incubated with control serum. Electron microscopy showed cytoplasmic inclusions in infected HL60 cells. The size of individual bacteria varied, and double-layered membranes were clearly observed surrounding electron-lucent and electron-dense forms (Figure 1, panel D).

### *A. phagocytophilum* Isolate Sequences

The 1,431-bp nearly entire *rrs* sequences of the 3 *A. phagocytophilum* isolates from cultured cells were identical to each other and to the sequences amplified from infected mice as well as from field-collected rodents and sheep. The tested *rrs* sequences were also identical to sequences amplified from ticks and rodents captured 3 years ago (GenBank accession nos. DQ342324 and DQ449948) in the same area (*8*) but different from all known *A. phagocytophilum* sequences deposited in GenBank.

**Figure 2:**
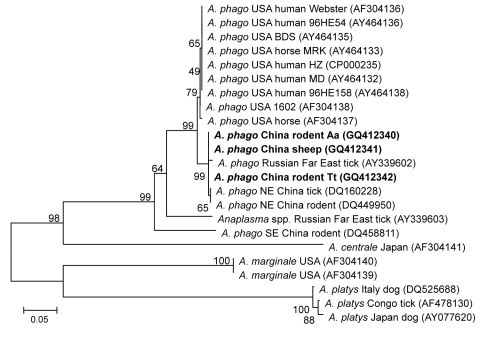
Phylogenetic tree based on partial (348-bp) *gltA* sequences of *Anaplasma* spp., obtained by using neighbor-joining method with Kimura 2-parameter analysis and bootstrap analysis of 1,000 replicates. Numbers on the branches indicate percentage of replicates that reproduced the topology for each clade. Parentheses enclose GenBank numbers of the sequences used in the phylogenetic analysis. **Boldface** indicates sequences obtained from rodents and sheep from northeastern China, May 2009. Scale bar indicates number of nucleotides per 1,000 bp. *phago, phagocytophilum.*

Analysis of the partial sequences of *gltA* (348 bp), *msp4* (779 bp), and *groESL* (428 bp) genes showed that the nucleotide sequences of *gltA* fragments amplified from the 3 isolates were identical to each other and showed 84% –99% identity with previously reported *A. phagocytophilum* strains, with 3–52 bp differences and 83%–99% similarity of deduced amino acid sequences. Three clades were structured on a phylogenetic tree based on 348-bp nt of the *gltA* gene, including a clade of strains from the United States, the Russian Far East, and this study; a clade comprising strains from rodents in southeastern China; and a clade of other *Anaplasma* spp., such as *A. centrale, A. marginale*, and *A. platys* (Figure 2).

**Figure 3:**
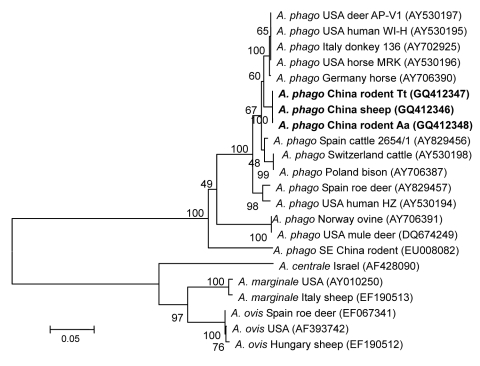
Phylogenetic tree based on partial (779-bp) *msp4* nt sequences of *Anaplasma* spp., obtained by using the neighbor-joining method with Kimura 2-parameter analysis and bootstrap analysis of 1,000 replicates. Numbers on branches indicate percent of replicates that reproduced the topology for each clade. Parentheses enclose GenBank numbers of the sequences used in the phylogenetic analysis. **Boldface** indicates sequences obtained from rodents and sheep from northeastern China, May 2009. Scale bar indicates number of nucleotides per 1,000 bp. *phago, phagocytophilum.*

The sequences of 779-bp *msp4* fragments amplified from the 3 isolates were also 100% identical and had 98%–87% nt sequence identity and 99%–88% deduced 268-aa sequence identity compared with *A. phagocytophilum* strains available in GenBank. When compared with the sequences from rodents in southeastern China (GenBank accession no. EU008082), nucleotide identity was only 87% with a 95-bp difference, and induced amino acid identity was 88% with a 31-aa difference. Phylogenetic analysis placed the *A. phagocytophilum* isolates in this study on a separate branch and in the same clade as the strains from the United States and Europe but far from the strains from sheep in Norway (GenBank accession no. AY706391), mule deer in Montana (DQ674249), and rodents in southeastern China (EU008082) (Figure 3). The other *Anaplasma* spp. were in a separate clade.

**Figure 4:**
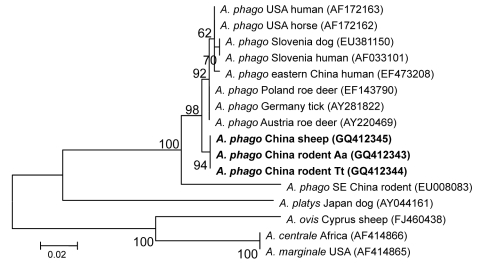
Phylogenetic tree based on partial (428-bp) *groEL* nt sequences of *Anaplasma* spp., obtained by using the neighbor-joining method with Kimura 2-parameter analysis and bootstrap analysis of 1,000 replicates. Numbers on branches indicate percent of replicates that reproduced the topology for each clade. Parentheses enclose GenBank numbers of the sequences used in the phylogenetic analysis. **Boldface** indicates sequences obtained from rodents and sheep from northeastern China, May 2009. Scale bar indicates number of nucleotides per 1,000 bp. *phago, phagocytophilum.*

When the 428-bp *groEL* sequences of the 3 isolates were compared with known *A. phagocytophilum* sequences in GenBank, the identity varied from 93% to 99%. The phylogenetic tree of *groEL* showed the *A. phagocytophilum* isolates in this study on a separate branch. The strains from humans in China and the United States, horses in United States, dogs in Slovenia, roe deer in Poland and Austria, and ticks in Germany were in another clade (Figure 4); however, their deduced amino acid sequences were identical to those from patients and rodents in southeastern China.

## Discussion

We isolated 3 strains of *A. phagocytophilum* from black-striped field mice, great long-tailed hamsters, and sheep in northeastern China. The availability of the isolates in a cell line will permit studies on the genetic, proteomic, and pathogenic characteristics of this agent.

*A. phagocytophilum* is reportedly maintained in various animal reservoirs, such as white-footed mice (*19*), woodrats (*7*), goats, sheep, and horses (*5**,**20*). Our isolation of 3 *A. phagocytophilum* strains from *A. agrarius* and *T. triton* rodents and from sheep indicates that both small wild animals and domestic animals may act as competent reservoirs of *A. phagocytophilum* in northeastern China. Although we found cultivation of this organism from experimentally infected mice to be reliable, the sensitivity of cultivation from wild and domestic animals is uncertain. In addition, the specimens used for isolation were pooled. Consequently, we were unable to ascertain the exact prevalence of infection in the rodents collected for this study. In a previous survey, we found a natural infection rate of 8.8% for *A. phagocytophilum* in rodents in the same area (*8*). To determine the level of infectivity in rodents as well as domestic animals, further studies are needed.

The nucleotide sequences of the 3 strains in this study were identical to each other in corresponding genes. The 1,431-bp nearly entire *rrs* sequences were most closely related to those detected in rodents from southeastern China (*8**,**9*), but they differed from other known strains. The sequence divergences and the phylogenetic analyses of partial *gltA*, *msp4*, and *groESL* genes indicated that a novel strain of *A. phagocytophilum* might be prevalent in northeastern China.

Different *A. phagocytophilum* strains seem to have special host tropisms (*21*). Strains from sciurids and white-footed mice infect various laboratory animals and perhaps humans as well. *A. phagocytophilum*–variant 1 and the strains from woodrats are found in association with wildlife only; human infections with these strains have yet to be identified. *A. phagocytophilum*–variant 1 has been unable to infect white-footed mice or SCID (severe combined immunodeficiency) mice but could infect goats by experimental inoculation (*22*). Holden et al. have documented that the pathogenicity of an *A. phagocytophilum* strain causing human disease waned with mouse passage in C3H mice but could be resurrected by passage in SCID mice (*23*). In our study, *A. phagocytophilum* strains with the same molecular characteristics were isolated not only from wild rodents but also from domestic sheep. Furthermore, they could propagate in BALB/c mice in the laboratory. The host tropisms and pathogenicity of the isolates remain to be clarified, and the relevance of these findings to public health and veterinary medicine deserves further investigation.
